# The Air That We Breathe: Addressing the Risks of Global Urbanization on Health

**DOI:** 10.1371/journal.pmed.1001301

**Published:** 2012-08-28

**Authors:** The PLOS Medicine Editors

## Abstract

The *PLOS Medicine* Editors discuss the need for transparent urban air quality indicators across the global and especially in low- and middle-income countries.

More than half of the world's population now live in cities [1], and while urbanization has the potential to allow greater access to health care for all, huge discrepancies in how resources are allocated within cities result in major inequities in health [2]. Addressing these discrepancies and improving health require accurate assessment. To that point, earlier this month *PLOS Medicine* published a Policy Forum article by Jason Corburn and Alison Cohen that focused on the urbanizing planet and the need for health equity indicators to guide public health policy in cities and urban areas [2].

The major theme of Corburn and Cohen's argument is that if societies are to ensure those living in the poorest urban slums have the same right to health as people living on the richest boulevards, health indicators must allow for the identification of where health inequities exist. For example, while indicators in Nairobi measure population access to communal toilet blocks, they give no information as to whether the toilet blocks are hygienic or safe to use and therefore mask inequity within the city. Such indicators, however, would have little value in cities like London or New York, which illustrates the need for context-specific measures.

There is a long history of public health interventions dramatically improving the health of a city. In London, both the Great Stink of 1858, caused by the River Thames becoming an open sewer [3], and the Great Smog of 1952, when London was shrouded in a dense smog for several days mostly due to burning coal as fuel [4], were tipping points that led to improved sewerage systems and clean air laws. These two examples were so extreme that it was not necessary to develop a metric to prompt action; the problems were there to be seen, breathed, and smelled. However, environmental problems should be tackled before they become overpowering; environmental hazards that are not obvious to residents still affect human health. Indeed, a recent study estimates that about 3,200 air-quality related deaths occur every year in greater London [5]. These more subtle hazards require indicators to assess and track their impact and progress.

The remaining air quality problems in London are dwarfed by those in cities without strong environmental laws and accountable government. Last year, World Health Organization (WHO) figures on the air quality of nearly 1,100 cities across the globe [6] found the worst air particulate matter not to be in the world's largest cities but from Iran (four cities), Mongolia, India (two cities), Pakistan (two cities), and Botswana—these ten cities have the highest mean annual levels of particulates sized 10 micrometers (µm) or less (PM10). For particulates of 2.5 µm or less (PM2.5), cities from Mongolia, Madagascar, Kuwait, Mexico, Ghana, Poland (two cities), Senegal, Italy, and Peru feature in the top ten.

WHO estimates that approximately 1.34 million premature deaths were attributable to outdoor air pollution in 2008 [6], with much of that burden falling within low- and middle-income countries. Examples of industrial exposure have demonstrated the devastating effect that prolonged exposure to high levels of respirable particulates can have on health [7] and, despite existing health and safety laws, occupational exposure to poor air quality remains a problem even in the richest countries [8]. The majority of outdoor airborne particulates in cities come from burning fossil fuels, and in particular from traffic and industry, and the practice of burning solid fuels for cooking and heating. A substantial body of evidence has demonstrated an association between exposure to particulates within the atmosphere, especially PM2.5, and adverse effects on health, with cardiovascular disease being the leading cause of air pollution–mediated morbidity and mortality [9].

The WHO figures show that only a few of the world's cities currently meet WHO guideline values for PM2.5 and PM10. Although the reported average values are high, the data don't include readings from industrial areas and other recognized “hot spots,” so the measurements are unlikely to be representative of the exposure endured by many individuals living in cities. Much of the data come from very few monitoring stations within a city and often from only one. Furthermore, although the data span 2003–2010, the great majority come from 2008–2009, making determination of trends difficult. Clearly there is a need for more air quality data from the world's cities—and in particular from those in low- and middle-income countries—but if those at highest risk within cities are to be identified, better local assessment seems warranted. Perhaps more worrying than inadequate measurement is when data, such as air quality, are seen by local and national governments as an embarrassment to be hidden away. Earlier this year China announced it would start monitoring PM2.5 and ozone levels in 27 provincial capitals and other key regions with the aim of expanding this program to all cities at prefecture level or above in 2015 [10]. While the statement, which set out the need for monitoring by stating, “we need firmer resolution and more effective measures, under higher standards, to remedy air pollution and steadily improve air quality,” is to be condoned, the reliability of the measures have been questioned [11]. Particularly concerning is the lack of concordance between the US Embassy's and the Ministry of Environmental Protection's measurements for PM2.5 in Beijing, with the US figures often being substantially higher than the official Chinese figures [12].

As Corburn and Cohen note in their article, for indicators to be effective they must be context-specific and relevant, and be made available transparently so that they can be open to interpretation and reevaluation. Only by measuring and disseminating such data will the inequities in the world's urban areas, and the steps necessary to address them, become clear.
